# Impact of *BRCA* Status on Reproductive Outcomes in Breast Cancer Patients in Romania: A Retrospective Study

**DOI:** 10.3390/diseases13090297

**Published:** 2025-09-08

**Authors:** Cristina Tanase-Damian, Diana Loreta Paun, Nicoleta Zenovia Antone, Alexandru Eniu, Carina Crisan, Eliza Belea, Anca-Magdalena Coricovac, Ioan Tanase, Patriciu Andrei Achimas-Cadariu, Alexandru Blidaru

**Affiliations:** 1 Department of Surgical Oncology and Gynecologic Oncology, Faculty of Medicine, “Iuliu Hatieganu” University of Medicine and Pharmacy, 8 Victor Babes Blvd, 400347 Cluj Napoca, Romania; damian.tanase.cristina@elearn.umfcluj.ro (C.T.-D.); nicoleta.antone@iocn.ro (N.Z.A.); beleaeliza@gmail.com (E.B.); pachimas@umfcluj.ro (P.A.A.-C.); 2 Department of Endocrinology, Faculty of Medicine, Carol Davila University of Medicine and Pharmacy, 8 Eroii Sanitari Blvd, 050474 Bucharest, Romania; 3 Breast Cancer Center, Institute of Oncology “Prof. Dr. Ion Chiricuta”, Str Republicii 34-36, 400015 Cluj Napoca, Romania; 4 European School of Oncology, Via Vincenzo Vela 6, 6500 Bellinzona, Switzerland; alexandru.eniu@hopitalrivierachablais.ch; 5 Hopital Riviera-Chablais Vaud-Valais, Grange de Tilles, 1847 Rennaz, Switzerland; 6 Department of Embryology, Faculty of Dental Medicine, Carol Davila University of Medicine and Pharmacy, 8 Eroii Sanitari Blvd, 050474 Bucharest, Romania; anca.coricovac@umfcd.ro; 7 Faculty of Medicine, Carol Davila University of Medicine and Pharmacy, 8 Eroii Sanitari Blvd, 050474 Bucharest, Romania; ioan.tanase@umfcd.ro (I.T.); alexandru.blidaru@umfcd.ro (A.B.)

**Keywords:** breast cancer, *BRCA*, pregnancy, fertility

## Abstract

**Background:** Breast cancer is the most frequently diagnosed cancer in women, and advances in genetic screening have led to a growing number of patients being identified as *BRCA* mutation carriers. For these women, the safety of pregnancy following cancer treatment remains insufficiently studied, and possible biological mechanisms—including defective DNA repair pathways and accelerated depletion of the ovarian reserve—may influence fertility potential and pregnancy outcomes. This exploratory research set out to examine whether *BRCA* status impacts reproductive outcomes in breast cancer survivors, while also considering underlying biological explanations for any observed differences. **Methods:** We performed a retrospective, single-institution cohort study involving young women with non-metastatic breast cancer who had undergone *BRCA* testing over a 17-year period. Clinical, oncologic, and reproductive data were collected and patients were followed longitudinally. **Results:** Of the 117 women who met eligibility criteria, 15 conceived at least once after cancer therapy; 11 carried no *BRCA* mutation, and 4 were *BRCA*-positive (2 with *BRCA*1 and 2 with *BRCA*2 variants). While the overall cohorts were broadly comparable, significant differences emerged in terms of tumor grade, hormone receptor status, *HER*2 expression, and treatment modalities. *BRCA* mutation status did not appear to influence reproductive outcomes, and all pregnancies in both groups progressed to full-term delivery without major obstetric complications or congenital anomalies. **Conclusions:** Within the limitations of a small, retrospective, single-center dataset without adjustment for confounding variables, these preliminary findings suggest that pregnancy after breast cancer may be safe for *BRCA* mutation carriers, with no apparent adverse effect on maternal prognosis or birth outcomes. Confirmation from larger, prospective, multicenter studies is essential to validate these results, clarify possible biological mechanisms, and inform evidence-based fertility counseling and survivorship planning for this patient population.

## 1. Introduction

Breast cancer is the second most common malignancy and the leading cause of cancer-related mortality in women under 40 years old [1]. Up to 12% of malignancies in this patient group occur in *BRCA* (Breast Cancer Gene) pathogenic-variant carriers [2]. Pathogenic variants in the *BRCA*1 and *BRCA*2 genes, belonging to the category of DNA double-strand-break repair genes, place female carriers at risk of developing several malignancies, of which breast or ovarian cancers are the most significant [3]. According to the National Comprehensive Cancer Network, more than 60% of women with a pathogenic germline mutation in *BRCA*1 or *BRCA*2 are projected to develop breast cancer over the course of their lifetime [4]. The breast neoplasia diagnosed in these populations is usually more aggressive than in *BRCA*-wildtype females, with *BRCA*1 mutation often being associated with triple-negative subtypes [5]. Breast cancer often occurs at a younger age in this population, frequently before parental project completion [6].

In recent years, there has been a growing number of studies focusing on *BRCA*-associated breast cancer. These studies cover molecular diagnosis, genetic testing, management of early or metastatic carcinoma, management of long-survival patients for their risk of second primary malignancy, surgical procedures, and clinical follow-up [7]. With improved life expectancy in females with *BRCA*-associated breast cancer [8], fertility and fertility preservation have become highly relevant topics in this specific oncological field.

Survival alone is no longer the standard of care in oncology in the 21st century; instead, successful reintegration into daily life should be the ultimate goal of multimodal treatment [9,10].

While pregnancy has been proven to be safe in women with breast cancer history overall [11,12,13,14,15], few data are available regarding a possible detrimental impact on the prognosis for the subset of patients carrying *BRCA* mutations [16].

Consequently, this study scrutinized the influence of the *BRCA* mutation status of breast cancer patients on reproductive outcomes in a population of Romanian women. To our knowledge, this analysis is among the first in Romania to investigate the impact of pregnancy on breast cancer outcomes in women carrying mutations in the *BRCA* germline while also reporting pregnancy, fetal, and obstetric outcomes.

## 2. Methods: Study Design and Patients

This retrospective cohort study was conducted at a single tertiary care center in Romania, focusing on women with *BRCA*-mutated breast cancer. Eligible participants were aged 40 or younger at diagnosis and had been treated for stage I to III breast cancer between 2000 and 2017. Only those with confirmed pathogenic germline mutations in *BRCA*1 or *BRCA*2 were included. The exclusion criteria encompassed individuals with *BRCA* variants of uncertain significance, a history of ovarian or other non-breast malignancies, noninvasive breast cancer, de novo stage IV disease, lack of follow-up data, or no post-treatment pregnancy information. Patients who were *BRCA* mutation carriers but had not developed breast cancer were also excluded. Ethical approval was obtained from the institutional review board, and all participants gave informed consent prior to inclusion. Clinical data were gathered with regard to tumor characteristics, treatment received, *BRCA* mutation type, reproductive outcomes, cancer recurrence, survival, and post-treatment pregnancies. All of the patients were monitored up to the present.

## 3. Objectives and Endpoints

The primary objectives of this study were to describe reproductive outcomes of the subsequent pregnancy for patients diagnosed with breast cancer and *BRCA* mutation. The collected data for subsequent pregnancies included age at conception, interval from breast cancer diagnosis, mode of conception (spontaneous or ART), pregnancy outcome, live births, gestational age (preterm (<37 weeks) or full-term (≥37 weeks)), complications (maternal, fetal, or delivery-related), and breastfeeding. Pregnancies concurrent with cancer diagnosis were excluded.

## 4. Statistical Analysis

Pregnancy, fetal, and obstetric outcomes were primary endpoints. Descriptive analysis was used to evaluate the two groups while considering the time interval between oncological diagnosis and the reproductive history, along with its particularities and outcomes.

All of the data from the study were analyzed using IBM SPSS Statistics 25 and formatted using Microsoft Office Excel/Word 2021. The Shapiro–Wilk test was used to test for the normal distribution of the analyzed quantitative variables, with these being written as averages with standard deviations or medians with interquartile ranges. Absolute values or percentages were used in qualitative variables, and differences between groups were tested using Fisher’s Exact Test. Z-tests with Bonferroni correction were applied for more precise results in the contingency tables.

Mann–Whitney U tests were used to test the quantitative independent variables with non-parametric distribution. Student’s *t*-tests were used to test quantitative independent variables with a normal distribution between groups. Survival analyses, including overall survival, disease-free survival, progression-free survival, and time from diagnosis to pregnancy, were conducted using the Kaplan–Meier curve. The differences in survival times between *BRCA* gene groups were determined with Tarone–Ware or Log-rank tests.

## 5. Results

One hundred and seventeen young patients diagnosed with breast cancer were eligible to be included in the current analysis, of whom fifteen had at least one pregnancy after breast cancer treatment; eleven were of the *BRCA*-wildtype and four were *BRCA*-positive (two patients were *BRCA*1-positive and two patients were *BRCA*2-positive). In our study, 4 out of 15 pregnant patients in both cohorts opted for induced abortion. The baseline patient, tumor, and treatment characteristics are listed in Table 1.

The age at diagnosis was not significant between groups (*p* = 0.421/*p* = 0.588). Most patients were aged between 31 and 35 or 36 and 40, with the median age being 34 in the *BRCA*-negative group and 35.5 in the *BRCA*1 group/34.5 in the *BRCA*2-positive group. Thirteen patients out of fifteen in the pregnancy cohort were younger than 35 at diagnosis. 

The histological subtypes were not significantly different between groups (*p* = 0.495), with most of the patients having a ductal carcinoma (59.3%—*BRCA*-negative group; 50%—*BRCA*1 group; 63.6%—*BRCA*2 group).

The tumor grade was particularly distinct between groups (*p* = 0.001), with *BRCA*1 patients being more associated with poorly differentiated tumors (75% vs. 30.3%).

Hormone receptor status was significantly different between groups (*p* < 0.001), with *BRCA*1 patients being more frequently ER- and PR-negative (85.7% vs. 18.9%/27.3%), while *BRCA*-negative patients or *BRCA*2 patients were more frequently ER- and/or PR-positive (81.1%/72.7% vs. 14.3%).

*HER*2 status did not differ significantly between groups (*p* = 0.052), with most of the patients being *HER*2-negative (73.3%—*BRCA*-negative group; 72.9%—*BRCA*1 group, 91.7%—*BRCA*2 group).

The usage of chemotherapy was not significantly different between groups (*p* = 0.228); most of the patients had chemotherapy (90.1%—*BRCA* negative group; 87.5%—*BRCA*1 group; 83.3%—*BRCA*2 group).

The usage of endocrine therapy was significantly different between groups (*p* < 0.001), with *BRCA*-negative patients having more frequent endocrine therapy than *BRCA*1 patients (83.3% vs. 28.6%), while *BRCA*1 patients had less frequent endocrine therapy (71.4% vs. 16.7%).

For patients with medical history characteristics grouped by *BRCA* gene existence (listed in Table 2), it was observed that usage of birth control pills was significantly different between groups (*p* = 0.004), with *BRCA*-negative patients more frequently using birth control (73.6% vs. 42.3%) while *BRCA*-positive patients used birth control less frequently (57.7% vs. 26.4%). Prior medical history was significantly different between groups (*p* = 0.034); *BRCA*-negative patients showed a stronger association with no medical history (97.8% vs. 88.5%), while *BRCA*-positive patients were more associated with other comorbidities (e.g., diabetes mellitus or systemic lupus erythematosus) (11.5% vs. 1.1%).

## 6. Reproductive Outcomes

The data in Table 3 show the pregnancy, fetal, and obstetric outcomes in the pregnancy cohort grouped by *BRCA* gene status. There were 15 pregnant patients analyzed, with 11 being *BRCA*-wildtype and 4 being *BRCA*-positive (2 patients were *BRCA*1-positive and 2 patients were *BRCA*2-positive). All of the analyzed tests had a low/very low significance value due to the small number of pregnant patients.

The results show the following:

- Differences in time from diagnosis to pregnancy were insignificant between groups (*p* = 0.337), with the median period being 6 years in the *BRCA*-negative group, 2.5 years in the *BRCA*1 group, and 4.5 years in the *BRCA*2 group. The pregnancy interval was also not significant between groups (*p* = 0.328); most of the patients in the *BRCA*-negative group reported a duration of more than 5 years between diagnosis and pregnancy (54.5%), while most of the *BRCA*1 patients and *BRCA*2 patients had less than 2 years from diagnosis to pregnancy (50%/50%), but the differences observed could not be proven to be significant.
- The pregnancy outcomes were not significant between groups (*p* = 0.292). Most of the patients had one live birth: 63.6%—*BRCA*-negative group; 100%—*BRCA*1 group; 0%—*BRCA*2 group).
- The timing of delivery was not significant between groups (*p* = 0.417). Most of the patients delivered the pregnancy at term (85.7%—*BRCA*-negative group; 50%—*BRCA*1-positive group).

For the rate of breastfeeding, a tendency towards statistical significance (*p* = 0.083) was observed in the direction of breastfeeding being more prevalent in the *BRCA*-negative group (85.7% vs. 0%), but the significance could not be demonstrated because of the limited number of analyzed patients, as the median duration of breastfeeding in the *BRCA*-negative group was 6 months (IQR = 1–18 months).

The data in Table 4 show the patient recurrence distribution grouped by the existence of the *BRCA* gene. The results show the following:

- The rate of recurrence was not significant between groups (*p* = 0.551). Most of the patients did not have any recurrence (81.7%—*BRCA*-negative; 92.9%—*BRCA*1 group; 100%—*BRCA*2 group).
- The rate of second primary malignancy was not significant between groups (*p* = 0.133). Most of the patients did not have any second primary malignancies (97.6%—*BRCA*-negative; 85.7%—*BRCA*1 group; 100%—*BRCA*-positive).
- The rate of second primary breast cancer was not significant between groups (*p* = 0.312). Most of the patients did not have any second primary breast cancer (92.7%—*BRCA*-negative; 92.9%—*BRCA*1 group; 81.8%—*BRCA*2 group).

## 7. Discussion

This exploratory investigation represents the first detailed analysis from Romania evaluating pregnancy safety in young women with breast cancer who carry a *BRCA* mutation. Across 117 patients diagnosed with non-metastatic disease over a span of 17 years, 15 conceived at least once following cancer therapy. Our preliminary results indicate that, in this small cohort, pregnancy after breast cancer did not appear to negatively impact maternal prognosis and was generally associated with favorable outcomes for the newborn. All participants were monitored continuously until the time of this analysis.

Breast cancer is one of the most common cancers in young adult women [11]. Many women diagnosed with breast cancer may still be interested in a future pregnancy, but a positive *BRCA*1/2 germline mutation can significantly impact reproductive decision-making due to its long-term implications, including a lifetime risk of breast and ovarian cancers, an autosomal-dominant condition, and preventative surgeries [12]. In addition, *BRCA*1/2 germline mutation leads to impaired DNA repair, accelerating oocyte aging and reducing the oocyte reserve by initiating oocyte apoptosis [3]. Moreover, young patients diagnosed with breast cancer will more often fall within the criteria for genetic testing or counseling guidelines, therefore testing positive for a *BRCA* mutation. Most young, fit patients, especially those with TNBC, will undergo chemotherapy as part of their treatment plan, further altering their reproductive options, principally in the absence of fertility counseling beforehand. Above all, there is a sociocultural phenomenon of postponing parenthood due to the rise in effective contraception and increases in women’s education, but delayed childbirth (having one’s first child after the age of 30 years) is known to be a risk factor for breast cancer [13].

EUROSTAT figures indicate a consistent upward trend in the age of first-time motherhood across the European Union, with the average reaching 29.3 years by 2018 [14]. Additionally, approximately 10% of women diagnosed with breast cancer before the age of 40 carry a *BRCA*1 or *BRCA*2 mutation [15]. These statistics suggest that many young women in this demographic may either postpone or have not yet initiated childbearing when faced with a cancer diagnosis. This is why we have to understand that larger studies are needed to ensure that pregnancy after breast cancer in patients with germline *BRCA* mutations is safe without apparent worsening of maternal prognosis. Although conflicting results can be employed, a large, statistically powered study previously demonstrated the safety of subsequent pregnancy in young breast cancer patients irrespective of estrogen-receptor status, but only in a select subgroup of early-stage breast cancer patients [17].

Age at diagnosis was not significant between groups, with the median age being 34 in the *BRCA*-negative group, 35.5 in the *BRCA*1-positive group, and 34.5 in the *BRCA*2-positive group; 13 patients of 15 in the pregnancy cohort were younger than 35 years at diagnosis. This can be attributed to selection bias due to the fact that the cohort included patients who were tested for *BRCA* mutations according to national guidelines. Lambertini et al. found that *BRCA*1/2 carriers who became pregnant after breast cancer were usually younger and had early-stage tumors without lymph node involvement [18]. This may reflect the “healthy mother effect”—women with better outcomes are more likely to try for a pregnancy [19]. Experts advise waiting at least two years after starting hormone therapy to finish treatment and catch early relapses [18] . In our study, differences in time from diagnosis to pregnancy were not significant between groups, with the median period in the *BRCA*-negative group being 6 years, while it was 2.5 years in the *BRCA*1 group and 4.5 years in the *BRCA*2 group. The pregnancy interval was also not significant between groups, with most of the patients in the *BRCA*-negative group having more than 5 years from diagnosis to pregnancy (54.5%), while most of the *BRCA*1 patients and *BRCA*2 patients had less than 2 years from diagnosis to pregnancy (50%/50%), but the differences observed could not be proven to be significant.

On the basis of the last results from the POSITIVE trial [17], no international guidelines advise against pregnancy in young women with breast cancer who completed oncological treatment [20,21]. As mentioned, 1 in 10 women with breast cancer diagnosed under 40 years carry a *BRCA*1/2 mutation [22], and among these patients, a higher-than-expected pregnancy rate was observed (19% in 10 years). Our results showed that the pregnancy interval was also not significant between groups; most of the patients in the *BRCA*-negative group had more than 5 years from diagnosis to pregnancy, while most of the *BRCA*1 patients and *BRCA*2 patients had less than 2 years from diagnosis to pregnancy (50%/50%). This may be due to the lower proportion of patients with hormone receptor-positive tumors and the younger age of the patients at diagnosis, and may also be attributed to prophylactic salpingo-oophorectomy being recommended as early as possible for this group.

A key concern for these patients is the risk of adverse pregnancy outcomes, such as congenital anomalies linked to previous gonadotoxic treatments; although most studies are reassuring, some suggest higher rates of preterm birth and perinatal complications in breast cancer survivors [23].

The effect of chemotherapy on the uterine arteries is certainly a path to be investigated, as this would provide valuable information for both obstetrics and embryo transfer during in vitro fertilization procedures. Our data show that all pregnancies in the cohort resulted in full-term deliveries, with no adverse events or congenital malformations being observed. However, other studies with larger cohorts revealed a preterm rate of 9.2% [18,24], which is similar to that expected in the general population (approximately 11%) [25], and a congenital anomaly rate of 1.8% [12], which is 3% in the general population [26]. Prophylactic bilateral mastectomy in the *BRCA*-positive group made breastfeeding impossible, but it is important to note the role of breastfeeding among women with a *BRCA*1 or *BRCA*2 mutation. Data from one study show a protective role of breastfeeding against *BRCA*1-mutated breast cancer, but there is no protection for those with *BRCA*2 mutations. However, women with a *BRCA* mutation should be informed of the benefits of breastfeeding in terms of reducing breast cancer risk [27].

In our study, 4 out of 15 pregnant patients in both cohorts opted for induced abortion. The reason for this procedure was that patients did not expect to become pregnant after their oncological treatment. However, we have to inform patients that safe and reliable options for contraception are available for women who do not wish to conceive.

While there is much research supporting these studies, many physicians remain concerned about a potential relapse of breast cancer in germline *BRCA* mutation patients in the setting of pregnancy. The POSITIVE trial results demonstrated the safety of subsequent pregnancy in patients with hormone receptor-positive breast cancer, but only for select subgroups of early-stage disease, and with a lack of long-term data. There is a lack of studies regarding pregnancy in patients with a positive germline mutation. Even so, recent studies demonstrated a similar long-term prognosis for *BRCA*-mutated patients compared with *BRCA*-wildtype cohorts. Therefore, similar guidelines and precautions regarding pregnancy should be applied to these groups. Of course, our study should be considered in the context of its limitations, which include the retrospective nature, some missing information on the course, and the relatively small number of patients included in both cohorts, with an impact on the statistical results. 

To conclude, larger, prospective, multicentric studies are needed in order to confirm the safety of pregnancy in *BRCA*-mutated breast cancer patients. The present analysis, although limited by selection bias and the small number of patients, did not associate *BRCA* mutation with a worse prognosis in the setting of pregnancy, nor did pregnancy outcomes seem to be affected by the *BRCA* status. These findings could stand as a pillar for further investigations and could have an important impact on healthcare providers involved in counseling young *BRCA*-mutated patients with breast cancer who are concerned about the feasibility and safety of future conception.

### Limitations

Among the study limitations, it is important to note that these results should be interpreted in relation to the selected patient population on which the study was based. Clinical practice in breast cancer management, access to *BRCA* testing, and fertility preservation techniques evolved substantially between 2000 and 2017, the period during which our cohort was treated. Surgical and systemic treatments for breast cancer shifted from more radical approaches in the 1990s to breast-conserving surgery, sentinel node biopsy, taxanes, targeted therapies, and genomic-guided decision-making in the 2010s, with corresponding improvements in outcomes. *BRCA* testing, which was initially restricted to research and limited by high costs, became more accessible after 2010, leading to improved identification of hereditary cases. Similarly, fertility preservation, which was virtually unavailable in the 2000s, was introduced in the oncological consultation in the 2010s, with oocyte cryopreservation and fertility counseling offered. These temporal changes may have influenced survival, reproductive choices, and the representation of *BRCA*-positive patients, and should be considered when interpreting our results. It should also be acknowledged that data on fetal and obstetric outcomes were collected exclusively from oncological medical records or patient surveys, so potential under-reporting cannot be excluded.

## 8. Conclusions

This retrospective study is the first in Romania to evaluate the reproductive outcomes in young breast cancer patients with *BRCA* mutations. Our findings offer preliminary evidence that pregnancy after breast cancer treatment does not appear to adversely affect maternal prognosis, even in *BRCA*1/2 mutation carriers. Although the statistical significance of the obtained results was limited by the small sample size, reproductive outcomes such as live birth rates, breastfeeding, and delivery timing were comparable between *BRCA*-positive and *BRCA*-negative patients.

Importantly, *BRCA* status did not correlate with increased cancer recurrence or adverse obstetric outcomes in this cohort. These results support the notion that pregnancy is feasible and likely safe after breast cancer in *BRCA* mutation carriers, reinforcing current international guidelines. However, larger multicenter prospective studies are warranted to confirm these findings and provide stronger evidence to guide clinical practice in fertility counseling and cancer survivorship care.

## Figures and Tables

**Table 1:** Baseline patient and tumor characteristics grouped by *BRCA* gene existence.

Characteristic	*BRCA* Negative No. (%)	*BRCA*1 Positive No. (%)	*BRCA*2 Positive No. (%)	*p*-Value
No. of patients	91 (77.8)	14 (12)	12 (10.3)	-
Median age at diagnosis, years (IQR)	34 (31–36)	35.5 (32.2–39)	34.5 (32–36)	0.421 *
Age at diagnosis, years				0.588 **
≤30	17 (18.7)	3 (21.4)	1 (8.3)	
31–35	43 (47.3)	4 (28.6)	7 (58.3)	
36–40	31 (34.1)	7 (50)	4 (33.3)	
Histology				0.495 **
Ductal carcinoma	54 (59.3)	7 (50)	7 (63.6)	
Lobular carcinoma	3 (3.3)	0 (0)	0 (0)	
Mixed ductal/lobular	0 (0)	0 (0)	1 (9.1)	
Invasive, not otherwise specified	22 (24.2)	4 (28.6)	2 (18.2)	
Other	12 (13.2)	3 (21.4)	1 (9.1)	
Missing	0 (0)	0 (0)	1 (8.3)	
Tumor grade				0.011 **
1	18 (20.2)	0 (0)	0 (0)	
2	44 (49.4)	3 (25)	3 (33.3)	
3	27 (30.3)	9 (75)	6 (66.7)	
Missing	2 (2.19)	2 (14.28)	3 (25)	
Tumor size				0.178 **
T1 (≤2 cm)	16 (19.3)	0 (0)	3 (50)	
T2 (>2 to ≤5 cm)	44 (53)	5 (83.3)	3 (50)	
T3 (>5 cm) to T4	23 (27.7)	1 (16.7)	0 (0)	
Missing	8 (8.79)	8 (57.14)	6 (50)	
Nodal status				0.271 **
N0	24 (28.9)	8 (57.1)	5 (45.5)	
N1	36 (43.4)	3 (21.4)	3 (27.3)	
N2–N3	23 (27.7)	3 (21.4)	3 (27.3)	
Missing	8 (8.79)	0 (0)	1 (8.3)	
Hormone receptor status				<0.001 **
ER- and/or PR-positive	73 (81.1%)	2 (14.3%)	8 (72.7%)	
ER- and PR-negative	17 (18.9%)	12 (85.7%)	3 (27.3%)	
Missing	1 (1.09%)	0 (0%)	1 (8.3%)	
*HER*2 status				0.052 **
Negative	66 (73.3%)	13 (72.9%)	11 (91.7%)	
Positive	24 (26.7%)	1 (7.1%)	1 (8.3%)	
Missing	1 (1.09%)	0 (0%)	0 (0%)	
Breast surgery				0.948 **
Conserving	50 (55.6%)	8 (57.1%)	6 (50%)	
Radical	40 (44.4%)	6 (42.9%)	6 (50%)	
Missing	1 (1.09%)	0 (0%)	0 (0%)	
Chemotherapy				0.228 **
No	7 (7.7%)	0 (0%)	2 (16.7%)	
Yes	82 (90.1%)	14 (87.5%)	10 (83.3%)	
Missing	2 (2.2%)	0 (0%)	0 (0%)	
Type of chemotherapy				0.003 **
Anthracycline- + taxane-based	47 (68.1%)	7 (50%)	1 (12.5%)	
Anthracycline-based	13 (18.8%)	6 (42.9%)	7 (87.5%)	
Taxane-based	2 (2.9%)	1 (7.1%)	0 (0%)	
Other	7 (10.1%)	0 (0%)	0 (0%)	
Missing	13 (15.85)	0 (0)	2 (20)	
Endocrine therapy				<0.001 **
No	15 (16.7)	10 (71.4)	3 (30)	
Yes	75 (83.3)	4 (28.6)	7 (70)	
Missing	1 (1.09)	0 (0)	2 (16.7)	
Type of endocrine therapy				0.089 **
Tamoxifen alone	10 (14.1)	0 (0)	0 (0)	
Tamoxifen and LHRHa	56 (78.9)	2 (50)	6 (100)	
LHRHa alone	1 (1.4)	2 (50)	0 (0)	
AI with LHRHa	2 (2.8)	0 (0)	0 (0)	
Other	2 (2.8)	0 (0)	0 (0)	
Missing	4 (5.33)	0 (0)	1 (14.28)	
Median duration of endocrine therapy in months (IQR)	60 (60–96)	34 (6.5–87)	63 (32.25–93)	0.385 *
Missing	4 (5.33)	0 (0)	1 (14.28)	
Breast cancer during pregnancy				1.000 **
No	84 (92.3)	13 (92.9)	11 (100)	
Yes	7 (7.7)	1 (7.1)	0 (0)	
Missing	0 (0)	0 (0)	1 (8.3)	

* Mann-Whitney U Test, ** Fisher’s Exact Test.

**Table 2:** Patients’ medical history characteristics grouped by *BRCA* gene existence.

Characteristic	*BRCA*-Negative No. (%)	*BRCA*-Positive No. (%)	*p*
Smoking habit			0.272 **
Never smoker	53 (58.2)	16 (61.5)	
Smoker	29 (31.9)	10 (38.5)	
Former smoker	9 (9.9)	0 (0)	
Age at menarche, years (IQR)	13 (12–14)	13 (11.87–14)	0.279 *
Use of birth control pills			0.004 **
Never used	24 (26.4)	15 (57.7)	
Prior use	67 (73.6)	11 (42.3)	
Number of children			0.734 **
0	23 (25.3)	6 (23.1)	
1	41 (45.1)	10 (38.5)	
2	26 (28.6)	10 (38.5)	
3	1 (1.1)	0 (0)	
Treatment for infertility			1.000 **
No	88 (96.7)	26 (100)	
Yes	3 (3.3)	0 (0)	
Prior gynecological surgery			0.406 **
No	84 (92.3)	24 (92.3)	
Unilateral oophorectomy	4 (4.4)	0 (0)	
Bilateral oophorectomy	1 (1.1)	0 (0)	
Any gynecological surgery without oophorectomy	2 (2.2)	2 (7.7)	
Prior medical history			0.034 **
No	89 (97.8)	23 (88.5)	
Endometriosis	1 (1.1)	0 (0)	
Others	1 (1.1)	3 (11.5)	

* Mann–Whitney U Test and ** Fisher’s Exact Test.

**Table 3:** Pregnancy, fetal, and obstetric outcomes in the pregnancy cohort grouped by *BRCA* gene existence.

Outcome	*BRCA* Negative No. (%)	*BRCA*1 Positive No. (%)	*BRCA*2 Positive No. (%)	*p*
No. of patients	11 (15.7)	2 (14.3)	2 (25)	-
Missing	21 (23.07)	0 (0)	4 (33.3)	-
Time from diagnosis to pregnancy, years (Median (IQR))	6 (3.5–8)	2.5 (2–3)	4.5 (1–8)	0.337 *
Pregnancy interval				0.328 **
≤2 years from diagnosis	1 (9.1)	1 (50)	1 (50)	
2–5 years from diagnosis	4 (36.4)	1 (50)	0 (0)	
>5 years from diagnosis	6 (54.5)	0 (0)	1 (50)	
Type of conception – Spontaneous pregnancy	11 (100)	2 (100)	2 (100)	-
Pregnancy outcome				0.292 **
One live birth	7 (63.6)	2 (100)	0 (0)	
Induced abortion	3 (27.3)	0 (0)	1 (50)	
Spontaneous abortion/miscarriage	1 (9.1)	0 (0)	1 (50)	
Timing of delivery				0.417 **
At term (≥37 weeks)	6 (85.7)	1 (50)	0 (0)	
Preterm (<37 weeks)	1 (14.3)	1 (50)	0 (0)	
Breastfeeding				0.083 **
No	1 (14.3)	2 (100)	0 (0)	
Yes	6 (85.7)	0 (0)	0 (0)	
Median duration of breastfeeding, months (IQR)	6 (1–18)	-	-	-

* Kruskal–Wallis H Test and ** Fisher’s Exact Test.

**Table 4:** Patient recurrence distribution grouped by *BRCA* gene existence.

Characteristic	*BRCA* Negative No. (%)	*BRCA*1 Positive No. (%)	*BRCA*2 Positive No. (%)	*p* *
Invasive breast cancer recurrence				0.551
No	67 (81.7)	13 (92.9)	10 (100)	
Loco-regional	5 (6.1)	1 (7.1)	0 (0)	
Distant	10 (12.2)	0 (0)	0 (0)	
Missing	9 (9.89)	0 (0)	2 (16.7)	
Second primary malignancy				0.133
No	80 (97.6)	12 (85.7)	11 (100)	
Yes	2 (2.4)	2 (14.3)	0 (0)	
Missing	9 (9.89)	0 (0)	1 (8.3)	
Second primary breast cancer				0.312
No	76 (92.7)	13 (92.9)	9 (81.8)	
Yes	6 (7.3)	1 (7.1)	2 (18.2)	
Missing	9 (9.89)	0 (0)	1 (8.3)	

* Fisher’s Exact Test.

## Data Availability

The data presented in this study are available from the corresponding author upon request. The data are not publicly available due to privacy restrictions.
